# Methadone Induced Sensorineural Hearing Loss

**DOI:** 10.1155/2013/242730

**Published:** 2013-07-29

**Authors:** Chadi Saifan, Daniel Glass, Iskandar Barakat, Suzanne El-Sayegh

**Affiliations:** Department of Medicine, Staten Island University Hospital, 475 Seaview Avenue, Staten Island, NY 10305, USA

## Abstract

*Background.* Sudden sensorineural hearing loss (SSHL) caused by opiate abuse or overuse has been well documented in the medical literature. Most documented case reports have involved either heroin or hydrocodone/acetaminophen. Recently, case reposts of methadone induced SSHL have been published. *Case Report.* We present the case of a 31-year-old man who developed SSHL after a methadone overdose induced stupor. He was subsequently restarted on methadone at his regular dose. On follow-up audiometry exams, he displayed persistent moderately severe sensorineural hearing loss bilaterally. *Discussion.* This case is notable because unlike all but one previously reported case, the patient—who was restated on methadone—did not make a complete recovery. *Conclusion*. Methadone overuse in rare cases causes SSHL.

## 1. Case Report

Mr. A. L is a 31-year-old man who was brought in by ambulance to the emergency department (SIUH South) after being found by his mother confused and stuporous at home. EMS reported that he was found apneic which resolved with naloxone. Mr. A. L has a known medical history of polysubstance abuse. He is also known to have depression and anxiety with recent admission to the psychiatric ward secondary to suicide attempt (wrist cut). He was tapered off benzodiazepines and started on fluoxetine. He had also recently been placed on a methadone program and follows with the methadone clinic for that matter. The patient arrived to the emergency department lethargic but awake and oriented x3 having received naloxone by EMS and was cooperative with the medical staff. His vitals on admission where remarkable for a heart rate of 110, a blood pressure of 88/50 mmHg, and a temperature of 101 F. He claimed that he took the entire weekend supply of methadone (80 mg methadone that was supposed to be divided to 40 mg on Saturday and 40 mg on Sunday) on the same day in an attempt to “feel high” and was not trying to “kill himself.” His only complaint was acute bilateral hearing loss. Work up in the emergency department revealed severe respiratory acidosis (pH = 7.2, CO_2_ = 57) with right side atelectasis on chest X-ray, acute renal failure (Cr = 3.1 baseline 1.15), possible NSTEMI, and acute hearing loss. He was given ASA 325 mg and was placed on IV fluids and bicarbonate, broad spectrum IV antibiotics, and heparin drip, given a dose of charcoal, placed on a 1 : 1 sitter, and admitted to the intensive care unit for further management and treatment.

On further questioning he stated that he had normal hearing up until the time that he had become stuporous. Immediately upon wakening and before receiving any medicines besides naloxone, he noticed that he had complete hearing loss bilaterally. He denied tinnitus or vertigo. Otoscopic exam was normal bilaterally.

The following day the patient was feeling better (mentally and physically), and his physical exam was unremarkable except for bilateral hearing loss which was still complete. Patient had leukocytosis of 14,000 and had a positive urine analysis, and he also spiked a temperature of 102 F that night while still on IV antibiotics. His d-dimer levels were elevated, and in the setting of hypoventilation, a lower extremity duplex was done and came back as negative. He was evaluated by critical care medicine, cardiology, toxicology, renal, and psychiatry. Patient was cleared by cardiology as his cardiac enzymes were trending down, and it was thought to be secondary to his acute kidney injury (to be sent home on ASA 81 mg PO daily along with atorvastatin and metoprolol and was asked to follow up as an outpatient with a stress echo). His renal function continued to improve with IV fluids (renal/bladder sonogram showed mild renal hydronephrosis. Urology was consulted and determined that there is no need for any intervention secondary to nonobstructing stone in patient with known history of nephrolithiasis versus methadone toxicity). Urine toxicology screen came back positive for methadone, benzodiazepines, and barbiturates, the latter two which he was supposed to have been detoxed off of recently. A CT scan of the chest was negative for pulmonary embolism. The decision by the critical care team was to continue the unit monitoring as he continued to spike fevers and have chills. Patient was restarted on methadone 40 mg daily.

On the third day of admission he stated that, subjectively, his hearing had returned to thirty percent of normal and he was able to understand shouted words. Subsequently, the patient was sent to the medical floor with the active problems as follows: UTI, aspiration pneumonia, and IV site cellulitis. Meantime his hearing was improving slightly daily, although he remained far from baseline. Patient remained in the hospital for four days after that. On the medical floor his course was unremarkable, his vitals were stable, and patient was afebrile and more ambulatory. His subjective report of his hearing was improving day by day yet was still profoundly impaired, and he was given an appointment to see an ENT as an outpatient. He was discharged after a full week of hospitalization on oral levaquin to follow up with cardiology and ENT and methadone clinic.

**Figure 1:**
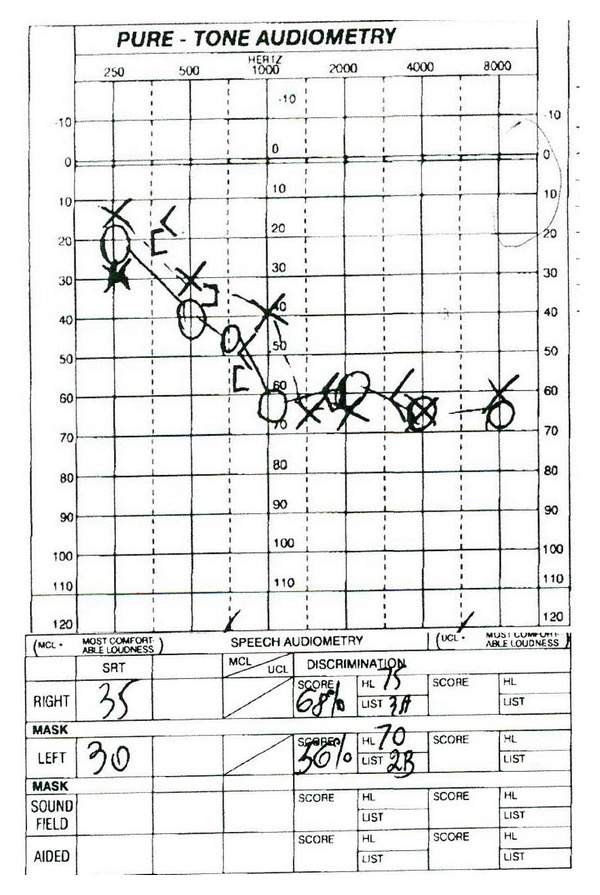
Audiometry graph one month after discharge.

**Figure 2:**
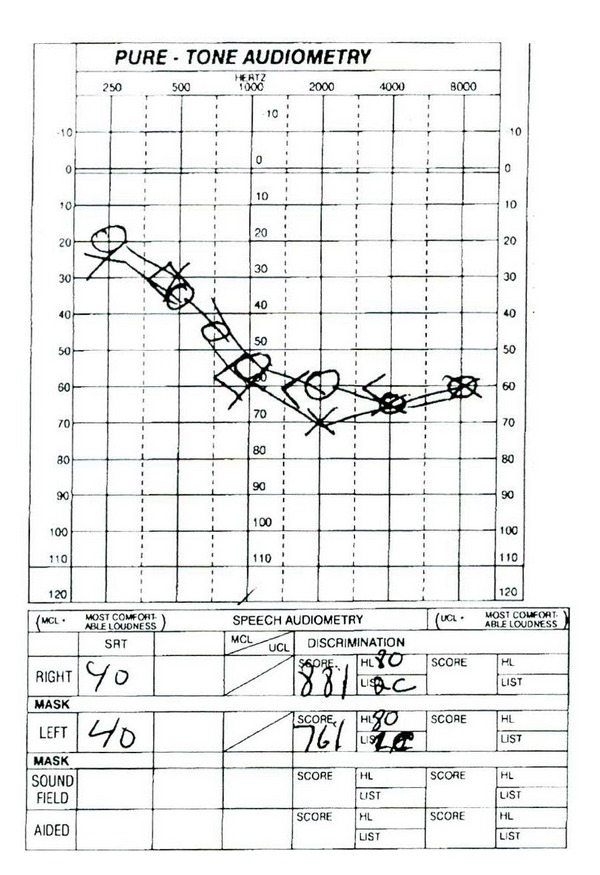
Audiometry graph two months after discharge.

Three weeks after discharge, patient followed with ENT and reported that his hearing was slightly improved yet still profoundly impaired. He denied vertigo although complained of tinnitus which was new. He underwent an audiology evaluation one week later (Figure 1) which revealed normal hearing at 250 Hz sloping to a moderately severe sensorineural hearing loss bilaterally with poor speech discrimination. Tympanometry revealed normal middle ear function bilaterally, and acoustic reflexes were present bilaterally except for an absent reflex in the left ear at 2,000 Hz. The exam was repeated one month later (Figure 2) with similar results, and he was prescribed binaural hearing aids. After this time, he was lost to follow up.

## 2. Discussion

This case presents a 31-year-old man who chronically uses methadone who developed multisystem failure and sudden sensorineural hearing loss (SSHL) following an acute methadone overdose. SSHL has no single definition but is generally considered hearing loss occurring over less than 72 hours of at least 3 contiguous frequencies with many cases occurring over minutes. It is a rare condition affecting about two patients per 100,000 person-years [5]. Bilateral involvement as in our case is a rare phenomenon. Some patients also describe associated tinnitus, vertigo, or aural fullness [5]. SSHL can be caused by a number of etiologies such as infections agents, otologic disease, trauma, vascular insult, neoplastic disease, and miscellaneous causes but with the majority of cases-being idiopathic [5]. Many of these “idiopathic” causes are caused by autoimmune processes [6, 7] or rarely, drugs, including antibiotics—classically aminoglycosides, diuretics, chemotherapies, and anti-inflammatory agents [3].

Numerous case reports have described SSHL following opiate abuse or overuse. Most cases have involved either heroin [8–14] or hydrocodone/acetaminophen [15–18] with varying degrees of recovery. Recently, four case reports have described acute SSHL in five patients upon awaking from coma or stupor induced by acute methadone overdose [1–4] (Table 1). Although it is difficult to prove causality of methadone for the SSHL, the time course is a compelling indication that methadone may be the cause. At least five of the six reported patients (including our case) received naloxone which improved the patient's level of consciousness which lends support that the altered level of consciousness was due to an opiod medication in exclusion to other medications that the patient may have ingested. As none of the patients who received naloxone were cogent and able to report potential hearing loss until receiving therapy, we are unable to determine whether naloxone may have contributed to the deafness.

The etiology of SSHL caused by methadone remains unclear. The theory that unknown substances contaminated into the drug are what cause the SSHL, which had been proposed for heroin-induced SSHL, seems dubious for methadone produced by professional laboratories. One author has proposed that SSHL caused by opiates may be partially due to a genetic predisposition but offers no evidence or elucidation [16]. As opiate-induced SSHL is extremely rare, to our knowledge no study has systematically attempted to understand the mechanism.

Although most cases of opiate-induced SSHL are thought to involve a retrocochlear process, one reported case of *chronic* hydrocodone/acetaminophen abuses led to sensorineural hearing loss that only improved after the insertion of cochlear implants [16]. The improvement with cochlear implants seems to suggest that the *chronic* hearing loss associated with *chronic* opiate abuse must be a cochlear rather than retrocochlear process. However, it is not clear whether we can extrapolate such a case to our case in which an *acute on chronic* methadone overdose induced *acute* hearing loss.

This paper adds to the growing body of the literature describing cases of SSHL secondary to methadone overdose. Our patient is only the second in the literature to not make a full and speedy recovery following methadone-induced SSHL. Why two of the six reported patients failed to make a full recovery is unclear. However, it should be noted that in our case the patient continued to receive methadone at his usually prescribed dose. In the other reported cases of persistent hearing loss, it is not known whether the patient continued methadone or not. Methadone was withheld in all of the reported cases of improvement in which data is available.

In summary, methadone overdose may in rare cases cause SSHL.

## Figures and Tables

**Table 1:** Review of published cases of methadone induced hearing loss.

Year	Authors	Age	Sex	Presentation	Naloxone given?	Substances Ingested	Associated conditions	Methadone withheld?	Followup
2009	Van Gaalen et al. [1]	37	M	Nausea, confusion. Deafness upon awakening	Yes	Methadone	Tinnitus, bradypnea	Yes	Complete resolution by day 10
2010	Christenson et al. [2]	30	M	Unresponsive and apneic. Deafness upon awakening in ED following naloxone	Yes	Methadone (large amount), cannabis, alprazolam and oxycodone (small amount)	Respiratory failure, ARDS, bradycardia, and Hypotension	Unknown	Resolution of hearing loss after 24 hours
2010	Christenson et al. [2]	25	F	Unresponsive. Deafness upon awakening	Yes	Methadone, citalopram, cannabis (all large amount), and oxymorphone (moderate amount)	Tachycardia, hypoxia	Yes	Mild improvement of hearing by 4 hours with complete resolution by 24 hours
2011	Shaw et al. [3]	20	M	Stupor following overdose. Deafness upon wakening	Yes	Methadone, alcohol, cannabis	Respiratory failure, leukocytosis, hypernatremia, AKI, and rhabdomyolysis	Yes	Complete resolution by day 4
2012	Vorasubin et al. [4]	23	M	Respiratory arrest. Deafness upon awakening	Unknown	Naltrexone, methadone	Respiratory failure	Unknown	Severe sensorineural hearing loss above 500 Hz persistent after at least 9 months
2013	Saifan et al. (this paper)	31	M	Apnea. Deafness upon awakening	Yes	Methadone, benzodiazepines, and barbiturates	Leukocytosis, fever, AKI, UTI, cellulitis, and aspiration pneumonia	No	Severe sensorineural hearing loss above 250 Hz persistent after at least 2 months
